# Including parents in inclusive practice: Supporting students with disabilities in higher education

**DOI:** 10.4102/ajod.v8i0.592

**Published:** 2019-10-21

**Authors:** Princess T. Duma, Lester B. Shawa

**Affiliations:** 1 Department of Human Resource Management, Mangosuthu University of Technology, Umlazi, South Africa; 2 Higher Education Studies, University of KwaZulu-Natal, Durban, South Africa

**Keywords:** family support, disability, South African universities of technology, inclusive education, forms of capital

## Abstract

**Background:**

While a number of research studies have endeavoured to understand students with disabilities’ experience in higher education and have recommended ways to effectively support student success, the role of parental support has been neglected. Many studies have been hampered by a limited understanding of students with disabilities and have, in particular, underestimated students’ ‘access to economic, social and cultural forms of capital’ that caring parents provide.

**Objectives:**

This article seeks to explore students with disabilities’ experiences of parental support in the South African higher education context. The research question guiding this article is: What forms of economic, social and cultural capital do parents and extended families provide to students with disabilities to enable them to succeed in higher education?

**Method:**

In-depth semi-structured individual and focus group interviews were conducted with 17 students with disabilities at two universities of technology. The interview transcripts were thematically analysed with a view to understanding Pierre Bourdieu’s forms of capital that parents provided.

**Results:**

The study found that while parents are not always able to provide material support, they offered rich and varied forms of social and cultural capital that enabled students with disabilities’ academic success.

**Conclusion:**

Given that parental support plays an important role in the success of students with disabilities, and this role changes as these students become more independent, this study recommends the need for universities to also pay more attention to involving parents in the education of the former. It is hoped that putting in place appropriate forms of parental involvement can create a conducive environment for universities to provide inclusive education holistically.

## Introduction

The transition from a basic schooling system to a tertiary institution often comes with mixed emotions for both students and parents. For students, it may mean independence as they emerge from the familiar home and school environment into the wider world of the higher education and experiencing the freedom of making one’s own decisions. According to Lane (2017),

all students both those with disability and those without, experience transitioning to higher education as stressful – new environments, new ways of learning and meeting new people is a rite of passage for millions of young people every year. (p. 18)

However, it has been widely reported that many students are underprepared academically for higher education studies, and this is associated with the high attrition and failure rate in South African universities. The DHET (2019) report reveals that some students take about a decade to complete a qualification as ‘68.8% (graduate) after 6 years of study and 78.8% (graduate) after 10 years of study’ (p. 30). According to Bannink, Idro and Van Geert (2016), some students become overwhelmed by academic demands and a sense of personal autonomy when they are away from their parents and a familiar environment. Pressure and confusion manifests itself among first-year students in particular when they need to acquaint themselves with the new environment, where they have to negotiate their social and academic spaces in an attempt to become productive members of the tertiary environment (Bonnani 2015). In this setting, the role of parents is relegated to a secondary position as students are expected to take charge of their studies and non-academic activities and this precludes parents (Bonanni 2015; Coccarelli 2010).

The institution enters into a contractual agreement with the student, regardless of whether the student understands it or not. Some students (first year in particular) may not understand several important issues, such as the manner in which the higher education system functions, for example how to deregister formally or what a fee increase entails. These processes deviate from their school experiences where parents were typically consulted. Moreover, universities’ operations and fees are sanctioned by the university council and not with parents (Edelman 2013).

Many students with disabilities come from special schools where environments are conducive for their particular conditions and needs (Bonanni 2015; Kelepouris 2014; Mcgregor et al. 2016). This is not the case at universities, as the extract from Daily Maverick (Van der Merve 2017) below reveals:

[*In special schools*] You have your teachers, just a few in the class, receive individual attention, and then you get to university and there are hundreds of you and nobody cares about you. (p. 1)

Leaving supportive learning environments in special schools behind is fraught with challenges for both students with disabilities and their parents. These challenges, with a specific focus on the role of parental support, are the focus of this article. It was pertinent and timeous to explore students with disabilities’ experiences of parental support in higher education, as their experiences could inform policy and practice within universities. Thus, the concern of the article is to explore the forms of economic, social and cultural capital that families provide which enable students with disabilities to succeed in their endeavours in a higher education setting.

### A brief overview of the literature on parental support for students with disabilities

The role of parents in higher education has attracted interest globally, and this has become evident in a growing body of literature (see Bethke 2011; Chadwick 2015; Edelman 2013; Garret 2015; Wong 2008). Currently, there are contradictory understandings on the importance of parental support to students. For example, Bethke (2011) and Chadwick (2015) found that parents’ involvement in the basic education of their children can be inappropriate if it is sustained in a higher education setting, although there are cases in which such support can have positive outcomes on student performance. While there is a large body of literature on the role of parents in the lives of children with disabilities in basic education in South Africa, there seems to be a dearth of literature on the significant influence of parental support on students with disabilities’ academic performance at university level (Esau 2018).

The literature suggests that there are appropriate and inappropriate forms of parental involvement (Touchette 2013). Wartman and Savage (2008) describe parental support as:

Parents showing interest in the lives of their students in college, gaining more information about college, knowing when and how to appropriately provide encouragement and guidance to their student connecting with the institution, and potentially retaining that institutional connection beyond the college years, (p. 5)

This description suggests that if parents are willing to work with universities, they could assist both their child and the university. It is natural for parents to care about the well-being of their children emotionally, financially, socially, academically and even spiritually (Chen & Ho 2012). Religion and spirituality are considered as another form of identity, which link to the cultural capital (Blanks & Smith 2009), as care is provided regardless of disability status of the child. Moreover, parents continue this support even when the child is enrolled at a tertiary institution (Edelman 2013; Garret 2015). In some instances, parents become ‘career counsellors’ who decide on the ‘perfect career’ and the appropriate institution for their children. Many children resent this kind of support, but it is difficult to negate parental decisions as parents may have experiences of tertiary institutions (Bethke 2011). Parental support is thus associated with a dependency effect which has merits and demerits, like in any other relationship (Edelman 2013; Garret 2015).

University structures tend to be intimidating and complex for new students; therefore, parental support in the selection of appropriate academic courses and registration processes are helpful (Edelman 2013). It is a common practice in South African universities to invite parents to an orientation briefing at the beginning of the year, but after this encounter there is no or little communication with parents. Moreover, the potential for a parent–university partnership has not yet been explored, probably because universities may wish to avoid parental interference at all costs (Kiyama et al. 2015). One view is that the influence of ‘helicopter parents’ needs to be eradicated in the interest of the students. In this regard, Vinson (2013) contends:

Helicopter parents hover from the prospective admissions stage to graduation and the job market beyond – contacting presidents of universities, deans, and professors, disputing their child’s grade; requesting an extension for their child; complaining their child does not receive as much praise as the parent would like; completing assignments for their child; requesting notification of grades their child received; and even attending job fairs and interviews with their child… (p. 423)

‘Helicopter parents’ tend to influence every stage of their children’s progress socially, pedagogically, and legally which is perceived as unprofessional, unfavourable and disruptive (Edelman 2013; Garret 2015; Haines 2017; Segrin et al. 2012).

Cullaty (2011) suggests that the role of parents should remain peripheral, where they should be supportive without meddling or intervening in their children’s university lives as students need to develop into responsible adults who can make their own decisions. This is because extreme parental support may have adverse effects on the development of students, thereby prolonging their transition to adulthood (Garret 2015). Touchette (2013) and Kelepouris (2014) argue that given the dynamics of global economics, the current generation of parents is more concerned with the future of their children compared with parents of the 20th century. Therefore, in response to the demand by parents for stronger involvement and support, some universities have launched programmes such as family weekends, parent orientations, family events on move-in day, parent newsletters, parent handbooks, parent associations and fundraising as an attempt to enhance parental involvement (Haines 2017). Well-designed programmes assist in building partnership between universities and parents for the benefit of the students and the university while also demarking the boundaries of parental support.

### Parental support

Parents of students with disabilities tend to be more involved with their children’s university life than most other parents as the challenges of the transition from high school to higher education are more demanding for these students (Lane 2017; Swart & Greyling 2011). Entry into higher education includes finding access to information (i.e. applying, finding an institution that best accommodates a specific disability and registering), finding suitable accommodation and choosing appropriate courses (Tugli et al. 2013). Adapting to a university’s demands depends on a number of factors such as character, social skills, nature of a disability, attitude, background and motivation (Strydom & Mentz 2010).

Various authors describe the barriers that students with disabilities encounter at university (Kendall 2016; Matshedisho 2010; Mutanga 2017). Lane (2017) broadly categorises these barriers as physical, attitudinal, social, cultural and political. Central to the challenges these students face are attitudinal barriers (Swart & Greyling 2011). For example, there seems to be a general lack of willingness on the part of some lecturers to provide the necessary support required by students with disabilities. Such an attitudinal position has an adverse impact on the academic performance of these students and, in some instances, even leads to failure or high dropout rate (Riddell, Wilson & Tinklin 2002). It is thus important that students, institutions of higher learning, parents and service providers co-operate and honour their responsibility of providing appropriate support to students with disabilities (Eckes & Ochoa 2005; Lang 2013).

Several recent studies have identified a number of specific areas in which parental support in the form of economic capital is of particular importance. For example, a recent study on the financial implications of disability identified three main areas in which students need financial support: (1) care and support for survival and safety, (2) accessibility of services and (3) participation in community activities (Hanass-Hancock et al. 2017). The latter study found that costs varied depending on the required care and support for the students as well as mandatory assistive devices such students need. Students with disabilities in South Africa are eligible as recipients of funds supplied by the National Student Financial Aid Scheme (NSFAS). However, accessing such funds is fraught with challenges (Bawa 2013; Lourens 2015; Ndlovu & Walton 2016). Parents and the families of students with disabilities may thus have to carry the financial burden to close the gaps when the funding scheme is lacking.

A strong cultural form of capital is prevalent among Africans that is associated with the spirit of Ubuntu (Taderera & Hall 2017; Walton 2018). Ubuntu is when people are not only concerned with their own well-being but help to address the needs of others too. Extended families are common in African culture, thus the absence of biological parents or their inability to adequately fund a child’s needs does not mean a student with disability will lack support, as family or siblings will often step in to ensure that the student is provided for (Williams 2011). As a supplementary supportive system, grandparents often become the caregivers when parents are busy, absent or deceased, although this support is not without challenges. Among the challenges that grandparents are likely to face are limited financial means, illiteracy and poor health (Bulanda & Jendrek 2016; Sampson 2015).

To date, the available literature reveals that there is a paucity of studies on parents’ and families’ support for students with disabilities in universities. The studies that could be traced tended to emphasise the role of the mother and generally find that ‘support provided by the biological fathers was minimal’ (Taderera & Hall 2017:8). Studies also found that students whose parents had received a university education had an advantage over ‘first generation’ in terms of support (Lorenzo & Cramm 2012; Williams 2011). The former group of students thus seems to be more likely to follow in their parents’ footsteps, as they understand the challenges that might be encountered in higher education settings. Moreover, these parents will have a better knowledge of social services and nongovernmental organisations (NGOs) that their children could access for additional support as they know that their families have aspirations for them (Gatlin & Wilson 2016).

Most students view academic success as a way of ‘paying back’ the investment made by parents. According to Chen and Ho (2012:317), it is a reciprocal relationship when ‘parents show their love by offering possible financial, material, and psychological support for learning, while the children return love by striving for academic excellence’.

Fuller et al. (2004) argue that both emotional and social forms of support were important for the academic success of students with disabilities. Many such students have a strong family culture that relies on prayer to support their academic endeavours (Kaye & Raghavan 2002). The literature suggests that while disability and student counselling units provide useful resources, emotional or spiritual support is more valued when it is obtained from those with whom students have a personal connection and who understand their backgrounds and personalities (Martinez 2015).

Students with disabilities, like most other students, create new images of themselves at university as they transform their self-images of vulnerability and dependency to that of capability independence and maturity. They soon view themselves as adults and soon-to-be professionals as they prepare themselves for the world of work (Darling 2013). Students tend to progress through various transitional stages towards emerging adulthood (Garret 2015), and they thus want to be viewed as capable, responsible and independent persons who can make their own decisions, regardless of their disability status. As their independence increases, they will no longer wish to be as dependent on parental support as before, and many even reject some forms of support (Kiyama et al. 2015). What parents should understand is that as their children reach new levels of independence and self-confidence, they should step back and engage in ‘less control and more communication’ (Fernández-Alonso et al. 2017:456).

## Research methodology

### Conceptual framework: Pierre Bourdieu’s forms of capital

The conceptual framework for this study draws from Bourdieu’s (1986) forms of capital as applied to higher education practices (Crozier et al. 2008; Yosso 2005) as well as to health and disability (Mithen et al. 2015; Pinxten & Lievens 2014). Bourdieu proposes three forms of capital: social, cultural and economic capital, and all three were deemed pertinent to university students with disabilities and the roles of their parents. Portes (1998:7) argues that ‘economic capital is in people’s bank accounts, cultural capital is inside their heads, and social capital [is] in the structure of their relationships’. The term ‘capital’ is typically understood as the financial resources that are available for purchasing goods and services; however, for Bourdieu there are additional symbolic forms of capital. For example, among the social groups that he studied, many valued strong neighbourhood ties, family bonds and social status as forms of capital or ‘wealth’. Bourdieu terms these elements ‘social capital’, and argues that some societies value social capital above economic capital. Cultural capital is another form of symbolic capital and describes the knowledge resources that an individual or group has accumulated. Cultural capital may also extend to religious beliefs and spirituality, which are seen as a symbol of hope across all communities (Blanks & Smith 2009). Such symbolic and abstract connections are likely to engender strong relationships, for instance between parents, children or between siblings.

People attend university to acquire particular forms of cultural capital, such as a professional knowledge and skills that can, in turn, be exchanged for economic capital. Bourdieu (1986:24) argues that the concept of capital is not necessarily limited to monetary value, but that ‘the forms of capital can be converted into other forms’, as in using cultural capital to acquire economic capital, using economic capital to buy books and thus gain cultural capital, or using social capital to progress in a workplace (and thus enhance economic capital). It is often more difficult for students with disabilities to acquire or ‘convert’ the social and cultural capital associated in higher education settings, and this can exacerbate the socio-economic disadvantage as they may find it difficult to acquire gainful employment (Mithen et al. 2015). It is thus all the more important for students with disabilities to draw on what Yosso (2005) calls ‘community [of] cultural knowledge, skills, abilities and contacts possessed by socially marginalised groups that often go unrecognised and acknowledged’ (2005:69). Therefore, by using these symbolic forms of capital, students with disabilities will gain maximum benefits from their higher education studies.

Unfortunately, a lack of application of Bourdieu’s forms of capital in education had the inadvertent consequences of making academic staff and administrators believe that disadvantaged students lack necessary forms of capital required for academic success, and this has, in some instances, encouraged ‘deficit thinking’ (Yosso 2005:69). Deficit thinking is the belief that students who do not succeed in their studies have personal deficiencies, that they are not intellectually capable of advancing or that they lack the motivation to learn. However, the application of Bourdieu’s theory could emphasise the resources that people have and not resources they lack (Pinxten & Livens 2014). Against this background, this article utilises Bourdieu’s theory as appropriate theoretical lens for exploring the issue of parental and extended family support for students with disabilities in the South African higher education context.

Researchers who draw on Bourdieu’s forms of capital make use of many different research approaches and methods, such as surveys, questionnaires, observations and interviews. Bourdieu himself used predominantly ‘ethnomethodology’ (Bourdieu 1986), which is an approach that included participant observation methods and extended in-depth interviews with research participants. These methods have enabled researchers to understand the life-worlds of the groups and individuals they have studied. Central to Bourdieu’s own research studies was a theorised understanding of the social groups and practices that he studied. Thus, his research was not ‘grounded’, but rather theoretically motivated and informed by forms of capital. For the purposes of the present study, individual and focus group interviews were conducted with a view to understanding the support that student participants received from their parents. Drawing on Bourdieu’s theory, the interview transcripts were thematically analysed and clustered according to the ‘forms of capital’ that emerged from the data.

### Sampling

Seventeen final-year students with disabilities participated in this study: 11 students participated in individual, semi-structured interviews and six participated in a focus group discussion. The final-year students were purposively sampled (Creswell 2013) with the assistance of the student counselling unit and disability unit at the two higher education institutions in KwaZulu-Natal province. The type of disability and programme of study were not the foci of the study as it concentrated on disability regardless of the type and intensity. The students were initially invited using emails and WhatsApp messages. The group was quite diverse in terms of gender and nature of their disabilities.

### Semi-structured interviews and focus group as data collection methods

Semi-structured interviews are commonly used in qualitative research for their strength in allowing the researcher to gain in-depth understanding of a phenomenon (Blandford 2013), which in this case was parental support for students with disabilities. To generate thick information and enhance the credibility of the study, a focus group discussion was also used to collect data. Six students with various forms of disabilities were invited to participate in the focus group. All ethical considerations for research of this nature were rigorously adhered to (Creswell 2009, 2013). Both data collection methods were aimed at exploring the students’ experiences of parental support, and the analysis of the data was underpinned by Bourdieu’s forms of capital.

### Ethical consideration

It is essential to adhere to ethical considerations when conducting research using representatives of a vulnerable group such as people with disabilities (Creswell 2013; Ramrathan, Le Grange & Shawa 2017; Ritchie & Lewis 2003; Yin 2011). The researcher thus adhered to the process for ethical approval as required by the selected universities of technology, and both granted permission for the study to proceed. The selected participants’ rights to confidentiality and to withdraw from the study at any point were explained to them, voluntary nature of their participation was emphasised and signed consent forms were procured. To adhere to confidentiality requirement, pseudonyms are used, while real names can only be accessed by the researcher.

## Findings and discussion: Parental support and ‘forms of capital’

The findings revealed that students had access to rich and diverse forms of capital as their parents and extended families were generally supportive of and committed to them. The findings are grouped in categories of (1) economic capital, (2) social capital and (3) cultural capital. There was considerable overlap across these groups, but the data findings are separated for analysis purposes.

### Economic capital

The first, and most obvious, form of capital that parents offered their children was economic capital in the form of financial support for their daily needs as well as for various other expenses such as a wheelchair or a motorcar. Most parents supplemented government disability grants and student bursaries. Economic capital thus includes all kinds of material resources that the students required.

#### From daily needs to major expenses

The data reveal how some students were financially dependent on their parents for their daily needs. Student 1 explained:

‘They [*parents*] support me in every way possible. I always lose my glasses and they would buy them for me and my glasses are very expensive.’ (Student 1, Mangosuthu University of Technology [MUT], female)

Student 2 mentioned:

‘After the accident my family bought me a car to make it easier for me to attend [*classes*] because I needed to heal completely before I could stay in the residence. Also, I did not qualify for NSFAS before the accident and it took time to get it after I became disabled.’ (Student 2, Durban University of Technology [DUT], male)

While non-disabled students generally find part-time employment in industries that typically employ students such as restaurants and shops, students with disabilities find it difficult to obtain part-time employment, either because of transport challenges or because of the physical nature of part-time work. A study by Majola and Dhunpath (2016) highlights the difficulty that people with disabilities face when they seek gainful employment. Most students were thus dependent on their parents for their everyday expenses as well as for the more expensive items.

#### Supplementing state-sponsored financial support

Most of the participants had access to economic capital through bursaries, study loans and disability grants like NSFAS. However, these funds were insufficient to cover the cost of living and needed to be supplemented by parents and families. Student 3 averred:

‘My mom and I had to put money together because I get a disability grant from the government. The university didn’t assist me, they knew about the situation from my first year. I’ve never been assisted with devices for my disability.’ (Student 3, DUT, female)

A student who participated in the focus group also found that the allocated budget was not sufficient:

‘Due to my visual challenge I have to change my glasses sometimes more than twice, my uncle assist me with this hence my parents cannot afford it. I tried to enquire from NSFAS office but could not be assisted in this regard.’ (Focus group, MUT, female)

Some students needed to use their bursary funds to buy medication or buy supportive devices, such as a wheelchair. The participants found that funding from NSFAS was helpful, but there were many delays in the system that retarded payment of the funds to the students, and this caused financial hardships. Bawa (2013) recorded a similar finding. In such cases parents had to make considerable sacrifices to assist their children. Most participants felt that automated wheelchairs would make their lives easier because they needed to move from their respective residence to other buildings just like any other student. Therefore, automated wheelchairs were considered to be a basic need. Unfortunately, many students were not able to afford a wheelchair as they are very expensive. Very few people can afford a device that costs about R30 000.00, and for these students this dream was unattainable. Parents who lacked economic capital because of low paying jobs or unemployment could not assist their children in this regard.

The financial contributions made by their parents were highly appreciated by the students, and they understood that without this economic capital support, they would have experienced even more difficulties in the pursuit of their studies. Several studies have also highlighted the impact of socio-economic status of parents on their children’s career (Ali et al. 2013; Esau 2018). One of the participants explained that his parents looked forward with great interest to his graduation ceremony; he understood that his academic success was his way of repaying the cost of his parents’ investment in his studies. Student 4 thus defined his graduation as follows:

‘the day when the investment matures.’ (Student 4, MUT, male)

### Cultural capital

Bourdieu (1986) proposes three kinds of cultural capital: (1) the institutionalised state (which refers to educational attainment), (2) the objectified cultural capital (this concerns the possession of cultural goods) and (3) the embodied or incorporated state capital (which refers to people’s values, skills, knowledge and tastes). It appeared from the participants that they benefited from the cultural capital that their parents had instilled in them, particularly in terms of spirituality and their sense of independence.

#### Spiritual support from parents

Spiritual support emerged as a very important aspect of support that the students had embraced. They revealed that spiritual support that their parents had instilled in them played an important role in sustaining their lives and therefore their studies. Student 3 explained:

‘I come from a prayerful family … parents always pray that their children become better people.’ (Student 3, DUT, female)

One participant in the focus group agreed with the importance of spiritual support:

‘When I finished high school in 2012. I was supposed to start university in 2013 and 2014 but unfortunately I felt very ill and could not start. So somewhere, somehow I lost hope and thought that may be education is not for me. But my mom prays a lot and encourages us to do so and she was like I shouldn’t give up because I’m still young and I can still do it.’ (Focus group, DUT, female)

The data revealed that these students had strong faith in God and believed that through their parents’ prayers, life would be better. They felt connected to their parents all the time. Prayer in this instance strengthened faith and hope so that the student felt secure and comforted, even in the face of adversity. Rule and Mncwango (2010 in Schoeman 2017) also found that around 63% of South Africans prayed several times a day. However, Blanks and Smith (2009) and Hartely (2004) found that religion and spirituality were not actively encouraged in higher education because of the wide diversity of religions that exist. Nonetheless, prayer was used as a motivating factor that propelled these students to work hard and succeed not only academically, but as courageous young people who had faced and were still facing many challenges. This finding resonates strongly with Bourdieu’s forms of cultural capital. While religion and spirituality are not directly actively encouraged by universities as observed by Blanks and Smith (2009) and Hartely (2004), students’ religious societies are allowed in most universities and students have a right to practise their religion of choice.

#### Parental aspiration as motivation for students to achieve

There are many ways of encouraging children to do well. Some need not to be conveyed verbally, but may be portrayed through the lifestyle standard that the family set, which could guide and motivate their children to do well in life. Student 9 described his family background as follows:

‘I think the standards they have set are too high both are educated, they are graduates. My mom has a degree in Social Science or Social Work I think. My dad has a Master’s degree in philosophy and had a red gown. They both graduated from the University of KwaZulu Natal.’ (Student 9, MUT, female)

Participants from the focus group also shared similar sentiments:

‘I grew up in a family that I can say everyone is highly educated, being that mom and aunt are teachers…’ (Student 5, DUT, male)

Another participant also mentioned:

‘I come from a home where people are studying even my mom is, my cousins and I also have a sister who was at Durban University of Technology in 2014.’ (Student 4, DUT, male)

Parental aspiration and educational level play a crucial role in academic performance of children (Chen & Ho 2012). Furthermore, Chen and Ho (2012:317) highlight the reciprocal relationship between the parents and their children. However, all or most parents have a vested interest in their children’s education and wish for them to succeed, especially when they will be the first in the family to achieve a university qualification. In this study, the majority of the participants came from households where the parents were well educated and worked as professionals. This status encouraged the students because their parents and other family members are their role models. In most cases parents understood the machinations of university life. According to Bourdieu (1986:244), ‘the scholastic yield from educational action depends on the cultural capital previously invested by the family.’

This kind of relationship is reciprocal as Chen and Ho (2012:317) mention that ‘parents show their love by offering possible financial, material, and psychological support for learning, while the children return love by striving for academic excellence.’

#### Students’ sense of independence

Some of the students seemed to have developed a very strong sense of independence and confidence in their own being. They agreed that all forms of support their parents wanted to give were welcome; however, their territory needed to be respected. Student 10 stated:

‘My independency has taken over my whole life. I do not like people doing things for me. No matter how sick I am I always find a way to do something.’ (Student 10, MUT, female)

This was echoed by Student 7:

‘We all have it in our minds that we can do things on our own but how if my parents starts coming to the university with me. Then it’s going to make me feel like different from other people.’ (Student 7, MUT, male)

Student 8 also cherished independence:

‘You know I did not involve anyone in the whole process of application and registration. At the beginning of the year I came here alone since I had a provisional offer. I was up and down trying to get information like anybody else until I was accepted. I went back home to take my stuff and I could not expect my granny to come with me from all the way from home to the university, as much as she wanted to. I assured her that I would be ok. I was just phoning her about everything because I knew she was worried.’ (Student 8, MUT, female)

It emerged from the data that some students did not want their parents to accompany them to university (Bethke 2011), as it might create the impression that they were struggling and were different from other students. These independent students wanted to eradicate the stereotypical thinking that people with disabilities are unfit to do things on their own. A previous study also found that the self-confidence and self-image of students with disabilities improved as they become more independent (Darling 2013).

At this level, students want to build a new image of themselves by changing their image of vulnerability to being perceived as capable, independent individuals as they prepare for the world of work. It is also important to acknowledge that these students are at a transitional stage, that of emerging adulthood (Garret 2015). They thus insisted that their own mode of understanding disability should change from charity model to social model. They did not want their parents to hover over their spaces as ‘helicopter parents’ who want to take over and lives of their children (Kiyama et al. 2015).

#### Communicating progress to parents

Although students felt that they needed space to manage their lives, they also had a sense of responsibility as they updated their parents on their progress. The data showed that they were willing to share their academic progress reports with their parents.

One participant from the focus group said:

‘Unfortunately I cannot send the results to her [*my granny*] because she is not educated. But when its holidays she does ask about how school was and whether I have passed or not.’ (Focus group, DUT, male student)

Student 4 stated:

‘I just usually screengrab my results for my mom because she lives very far and I only get to see her in December. However she is updated with everything for instance when I write tests or do presentation she knows. I have even given her my student portal password.’ (Student 4, MUT, male)

Student 2 offered the following:

‘My parents are supportive and always call to check how my exams went and the results. My results are posted and they do not wait for me to open it and I am happy with that since there is nothing to hide. They deserve to know anyway.’ (Student 2, DUT, male)

The communication channels described above seemed important in strengthening the support the students required. One participant even mentioned that he would be happy if the university had direct communication with the parents. The students were transparent and wanted to be trusted and supported, but from a distance. This not only ensured important social connections but also gave them the freedom to manage their lives.

### Social capital

Parents are generally key members of the social network and play a prominent academic role in the lives of all students (Ferrara 2015). While they might not always be able to assist their children financially or academically, they can offer forms of social support. Although some parents of participants did not have extensive business or professional networks, they were nevertheless able to provide considerable material care and moral support to their children.

#### Commitment and sacrifice: The wealth of mothers

Mothers played a particular role in ensuring the well-being of their children. The following extract describes the support and care Student 3 received from her mother:

‘In my first year she [*my mother*] used to come here to make sure I could attend classes. You know we attend in 3 different campuses Steve Biko, Ritson and ML Sultan campus. The challenge was I did not have an automated wheelchair, and could not wheel myself whole day. So she would come all the way from Inanda to push me around wherever my lesson was at the time. As you may know lessons are separated by 10 min if we started at Steve Biko in 10min we have to be at ML Sultan. Again sometimes there are intervals, where you don’t have a lesson in between or the lecturer is absent, she will wait with me. She did this for three months until she could afford to buy me a second hand [*automated*] wheelchair.’ (Student 3, DUT, female)

Not only did Student 3’s mother support her child by literally ensuring that she was able to get to her classes, but she managed to accumulate funds to purchase an automated wheelchair that helped her child to become more independent. Such extensive and compassionate maternal support was not uncommon among the interviewees. Student 6 shared the following:

‘My mom had to take a month leave in order to support me after the accident to see to it that I was adjusting well to my new status of disability.’ (Student 6, DUT, male)

Student 8 explained how her mother assisted her with childcare:

‘My mom has done a lot for me, she is even looking after my two year old son whilst I am at varsity. She takes him to a day care without which I would not be here.’ (Student 8, MUT, female)

The care and support offered by mothers is a rich source of social capital, and it was valued by the students, and without it, they would not have been able to succeed in their studies. The participants did not refer much to the role their fathers provided; in some cases the father was referred to as deceased or not taking responsibility, which is consistent with the finding by Taderera and Hall (2017).

#### The extended family: A support network

Many students had access to a wider social network comprising family members and friends, and the latter included residence roommates and peers. Some participants’ parents were deceased or not able to support them because of poor health. In such instances other family members supported them, as Student 6 explained:

‘Since my mom is not well; my brother has been a pillar of support. … He is my mentor we talk about everything more especially as I am at the university since he understands university life and challenges. He always give me good advices and sometimes I call him if something I do not understand happens or may be when I feel pressure with my tests. He always listens and sometimes just laughs at me.’ (Student 10, MUT, female)

Student 9 had a grandmother to offered unstinting support:

‘For me it is quite complicated. Both my parents have passed on, my grandmother is not educated but she tries everything within her capability to assist me.’ (Student 9, MUT, female)

The spirit of Ubuntu was clear in cases where orphans were able to pursue their studies with the assistance of grandmothers (Sampson 2015) and extended families. This spirit is based on a culture of taking care of others, and not only of blood relatives. The significance of the interplay of Bourdieu’s ‘forms of capital’ becomes evident when helping an orphan; it has a social capital, economic and cultural capital impacts.

#### Emotional and practical support

Most of the students admitted that it would have been difficult to cope without the emotional and practical support of their parents (or supportive others). Knowing that your parents were consistently supportive and were always available was important for Student 5.

‘My parents are supportive and always call to check how my exams went and the results. I remember one day I was panicking because my duly performed (permission to write exams) was very low for a certain subject. I had explained that to my mom because we talk about everything. On the day of examination she called in the morning she could feel that I was crying. I was much stressed she calmed me down and encouraged me, saying that I have worked so hard thus far and this time around I will make it again. You know what, I passed that module with 60 per cent!’ (Student 5 DUT, male)

Some focus group participants confirmed that while the emotional support of parents was important, they sometimes needed to be ‘selective’ in what they shared:

‘I would say emotional support is very important, especially when it comes to your academics. In varsity we go through a lot, you meet a lot of different people, from different backgrounds and you might want to tell your parent about all the stuff you are going through. But they might not understand so you then become selective in what you share with them. And it may become difficult to cope when you do not have any emotional support from parents.’ (Focus group, MUT, female)

While students appreciated the support they received from their parents and acknowledged their contribution to their academic success, it also became clear that they preferred not to completely share all of their challenges with their parents. The students wanted to protect their parents from some of the distress that they were experiencing, but they also did not want their parents to feel that they were not coping with university life. It underscored the reciprocal nature of social capital.

The students also did not refer to support offered by disability or student counselling units. This finding is supported by the findings of Martinez (2015), who found that students with disabilities benefited more from personal and familial contacts than from institutional support.

## Conclusion

Drawing on Bourdieu’s forms of capital as theoretical lens, this article has reported on a study that explored forms of economic, social and cultural capitals that parents and extended families provide to students with disabilities to enable them to succeed in two higher education settings. The study found that while parents struggled in the economic capital sphere as it was costly to provide expensive items such as automated wheelchairs and other assistive technologies, they were often able to assist with more basic requirements and to supplement state provisions. The study also found that parents and extended families were able to provide rich and varied forms of cultural and social capital. For example, while economic capital was necessary for these students with disabilities to cope with the challenges they faced, it was generally the cultural and social capital that their mothers provided that formed the basis of their of their support. This article also suggests that universities of technology in South Africa should explore the potential of parental support for students with disabilities.
